# Performance of a prototype electric silicone oil injector and aspirator in vitreoretinal surgery

**DOI:** 10.3389/fmed.2026.1855415

**Published:** 2026-06-02

**Authors:** Dehua Qin, Yali Nan, He Zhu, Xueying Wang, Yizong Liu, Ruiyan Fan, Dongdong Wang, Handong Dan, Hongling Chen, Zongming Song, Zixu Huang

**Affiliations:** 1 Department of Ophthalmology, Henan Provincial People’s Hospital, People’s Hospital of Zhengzhou University, Zhengzhou, China; 2 Henan Eye Hospital, Henan University People’s Hospital, Zhengzhou, China; 3 Henan Eye Hospital, Henan Medical University, Henan Provincial People’s Hospital, People’s Hospital of Zhengzhou University, Zhengzhou, China

**Keywords:** *ex vivo* porcine eye model, ophthalmic surgical instruments, silicone oil injection and aspiration, surgical performance, vitreoretinal surgery

## Abstract

**Aim:**

To develop a reusable prototype electric silicone oil injector and aspirator and to compare its performance with that of a vitrectomy machine-based system and a manual syringe technique in an *ex vivo* porcine eye model.

**Methods:**

The prototype device was designed with an optimized motor, control system, and human-machine interface to provide adjustable and stable silicone oil injection and aspiration in a compact portable format. Thirty fresh *ex vivo* porcine eyes were randomly assigned to three groups (electric injector, vitrectomy machine-based system, and manual syringe; *n* = 10 per group). Injection time, aspiration time, injection volume, and aspiration volume were recorded. Handling stability during injection was assessed by video-based shake-count analysis in a subset of procedures with analyzable recordings (*n* = 8 per group).

**Results:**

Injection time differed significantly among the three groups (*F* = 24.246, *p* < 0.001), with the manual group showing the shortest time (65.90 ± 15.34 s), whereas no significant difference was observed between the electric and vitrectomy groups. Injection volume did not differ significantly among groups (*F* = 0.676, *p* = 0.517). Aspiration time also differed significantly (*F* = 9.560, *p* = 0.001), with the electric group showing the shortest time (149.30 ± 53.63 s). Aspiration volume did not differ significantly among groups (*H* = 5.091, *p* = 0.078). For handling stability, the number of instrument shakes differed significantly among groups (*H* = 16.749, *p* < 0.001), with the manual group showing more shakes (42.0, IQR: 9.0) than both the electric (5.0, IQR: 1.5, *p* < 0.001) and vitrectomy group (8.0, IQR: 2.5, *p* = 0.014), while no significant difference was observed between the electric and the vitrectomy group (*p* = 0.769).

**Conclusion:**

Under the tested experimental conditions, the reusable prototype electric silicone oil injector and aspirator showed favorable aspiration performance and handling stability. Although its injection efficiency was lower than that of the manual technique, it was comparable to that of the vitrectomy machine-based system.

## 1 Introduction

Silicone oil (SO) is an important intraocular tamponade agent in vitreoretinal surgery, particularly for the management of complex retinal detachment, proliferative diabetic retinopathy, intraocular tumor and other advanced vitreoretinal disorders (1–6). Its clinical value in the repair of complex retinal condition has been further supported by recent multicenter clinical studies (6, 7, 41). Because SO is highly viscous, its injection and removal require precise control during surgery. Inadequate control may prolong surgical manipulation and contribute to incomplete oil removal, unstable intraocular pressure, or iatrogenic tissue stress (8–11)^.^

Despite its widespread use, currently available methods for SO injection-aspiration have notable limitations. Manual syringe-based techniques are simple and inexpensive, but they can be labor-intensive and less reproducible, particularly when high-viscosity SO is used (8, 11). Furthermore, disposable kits connected to vitrectomy machines may provide more stable delivery, but they depend on complex surgical platforms and can increase per-procedure material expenditure (12). In addition, SO-related complications, including emulsification and residual intraocular oil, remain important clinical concerns (5, 9).

These limitations highlight the need for a more practical and controllable SO injection–aspiration system that can reduce reliance on large vitrectomy platforms while maintaining stable intraoperative performance. To address this need, automated or motor-assisted approaches to viscous intraocular fluid exchange have already been explored (10, 11). Building on this rationale, our group developed a reusable prototype electric silicone oil injection–aspiration device incorporating a motor-driven actuation system and control interface designed to allow adjustable and consistent SO injection-aspiration. However, its comparative performance relative to conventional approaches has not yet been systematically evaluated.

Fresh *ex vivo* porcine eyes are widely used for the preliminary assessment of ophthalmic surgical instruments because of their anatomical similarity to human eyes and their suitability for posterior segment surgical simulation (13–15). Therefore, the present study aimed to compare the performance of this prototype with two commonly used approaches—a vitrectomy machine–based system and a manual syringe technique—in an *ex vivo* porcine eye model. The primary objective was to evaluate injection-aspiration performance and handling stability under controlled experimental conditions, thereby providing preliminary evidence for further device refinement and future *in vivo* investigation.

## 2 Materials and methods

All procedures involving porcine ocular tissues were performed in strict accordance with the Association for Research in Vision and Ophthalmology (ARVO) Statement for the Use of Animals in Ophthalmic and Vision Research. The fresh *ex vivo* porcine eyes used in this study were obtained as by-products from a local abattoir, with no animals specifically raised or euthanized for experimental purposes.

### 2.1 Experimental instruments and materials

#### 2.1.1 Experimental instruments

##### 2.1.1.1 Prototype electric silicone oil injector and aspirator

The prototype electric silicone oil injector and aspirator was developed to enable controlled silicone oil injection-aspiration during vitreoretinal procedures. The system consists of a handheld unit and a foot-operated control module. The handheld unit incorporates a high-precision micro linear motor and a dedicated control system designed to provide adjustable and stable actuation during both injection and aspiration process. The device was designed for single-handed operation to facilitate intraoperative handling.

The housing materials of the handheld unit were selected to tolerate steam sterilization at 135 °C, allowing repeated use under standard operating room sterilization conditions. The instrument interface was designed to be compatible with standard silicone oil injection-aspiration accessories. A compact foot control module was used with operator interface to allow activation and parameter control during the procedure.

**Figure 1:**
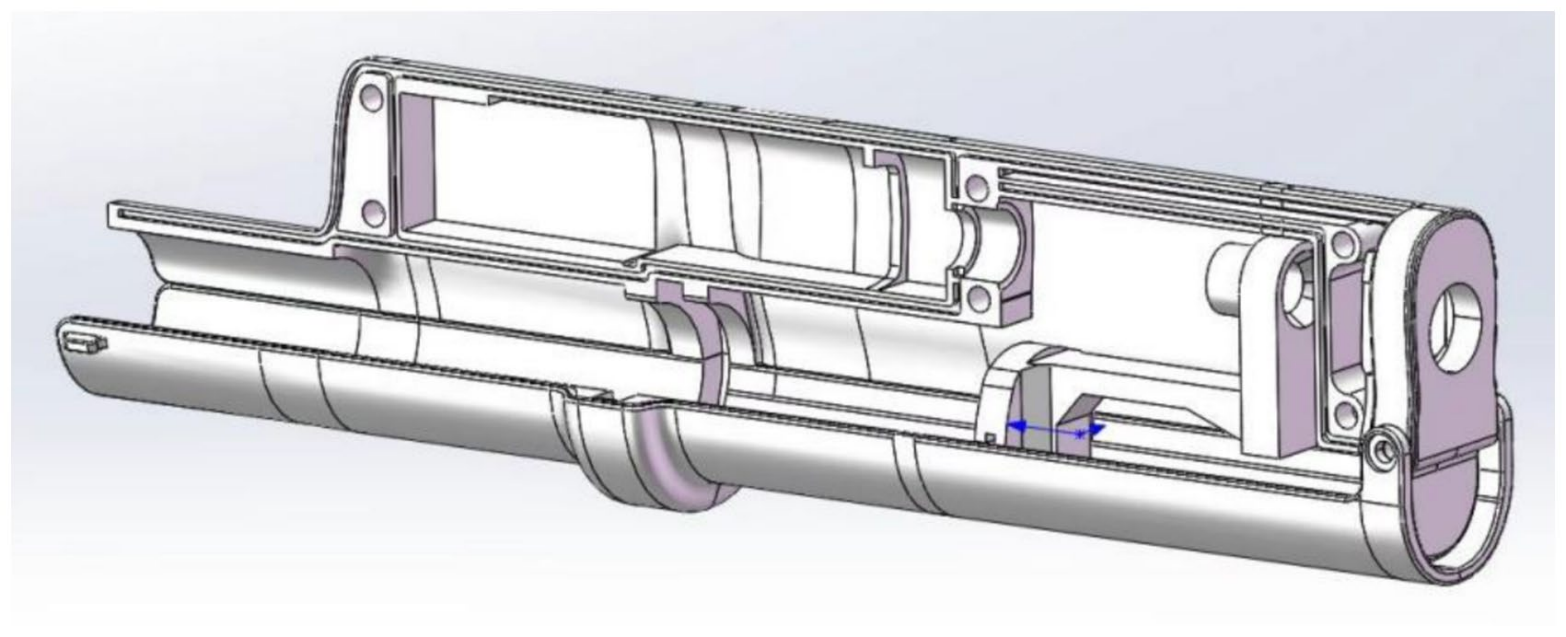
Schematic diagram of the handheld structure. Micro linear motor and SO injection/aspiration instruments were contained in handheld portion.

**Figure 2:**
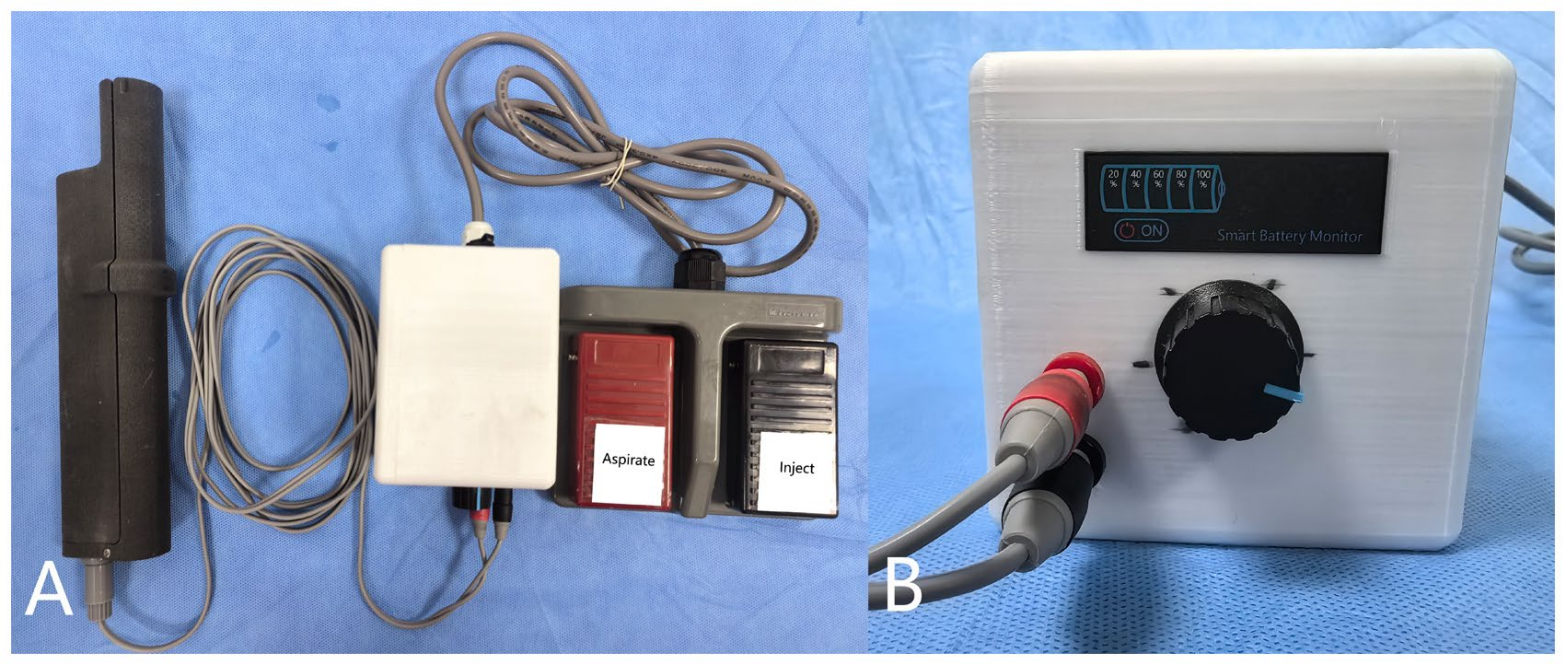
Photographs of the prototype. **(A)** Overall view: handheld portion, control module, foot pedal. **(B)** Control module with Variable from 1 to 5.

Figure 1 shows the schematic design of the handheld structure, including the micro linear motor and cavity for SO injection/aspiration instruments. Figure 2 presents photographs of the prototype system (A), and the control module (B).

##### 2.1.1.2 Vitrectomy machine and auxiliary instruments

A commercially available vitrectomy system (Bausch and Lomb, Stellaris PC, United States) equipped with its dedicated silicone oil injection-aspiration kit was used as the machine-based comparator. Additional instruments included a 23-gage vitrectomy cutter and related accessories, an operating microscope, a surgical video recording system, a screw-driven manual SO injector, and a digital timer with a resolution of 0.01 s. For the manual comparator group, 10-mL (injection) and 30-mL(aspiration) syringes fitted with matched 23-gage injection–aspiration needles were used.

#### 2.1.2 Experimental materials

Thirty fresh *ex vivo* porcine eyes were obtained from a local abattoir within 2 h after slaughter. After collection, the eyes were rinsed with sterile normal saline, stored at 4 °C, and rewarmed to room temperature for 1 h before use. Before inclusion in the experiment, each eye was examined under slit-lamp microscopy to exclude gross abnormalities that might interfere with the procedures, including marked corneal opacity, globe rupture, lens dislocation, or other visible structural damage.

Medical-grade silicone oil (5,000 cSt) was used in all experiments. Sterile normal saline was used for specimen preparation and surgical procedures. All eyes were handled according to the same preparation protocol to minimize variation related to postmortem tissue condition.

### 2.2 Experimental grouping and design

This was a comparative *ex vivo* study using 30 porcine eyes. The eyes were allocated to three experimental groups randomly, with 10 eyes in each group: (1) the prototype electric silicone oil injector and aspirator group (electric group), (2) the vitrectomy machine-based silicone oil injection/aspiration group (vitrectomy group), and (3) the manual syringe group (manual group). Group allocation was performed using a computer-generated random sequence.

To reduce operator-related variability, all procedures were performed by the same vitreoretinal surgeon with more than 5 years of surgical experience, assisted by the same surgical assistant throughout the study. All experiments were conducted on a standard ophthalmic operating table under an operating microscope. The entire injection and aspiration process was recorded using a surgical video system for subsequent assessment of handling stability.

The primary performance outcomes were injection time, aspiration time, injection volume, aspiration volume, and handling stability during the injection phase. Because this was an *ex vivo* comparative study, the results were intended to provide preliminary evidence regarding device performance under controlled experimental conditions rather than direct evidence of clinical safety or superiority.

### 2.3 Experimental methods

Each porcine eye was secured in a dedicated eye holder before surgery. After routine surface disinfection and sterile draping, three scleral entry sites were created 3.5 mm posterior to the limbus using 23-gage vitreoretinal instruments to establish a standard three-port configuration. A complete pars plana vitrectomy was then performed to remove the vitreous as thoroughly as possible, followed by air–fluid exchange to evacuate the intraocular fluid and create a standardized air-filled vitreous cavity before silicone oil injection.

#### 2.3.1 Silicone oil injection

For silicone oil injection, different devices were used according to group assignment.

**Figure 3:**
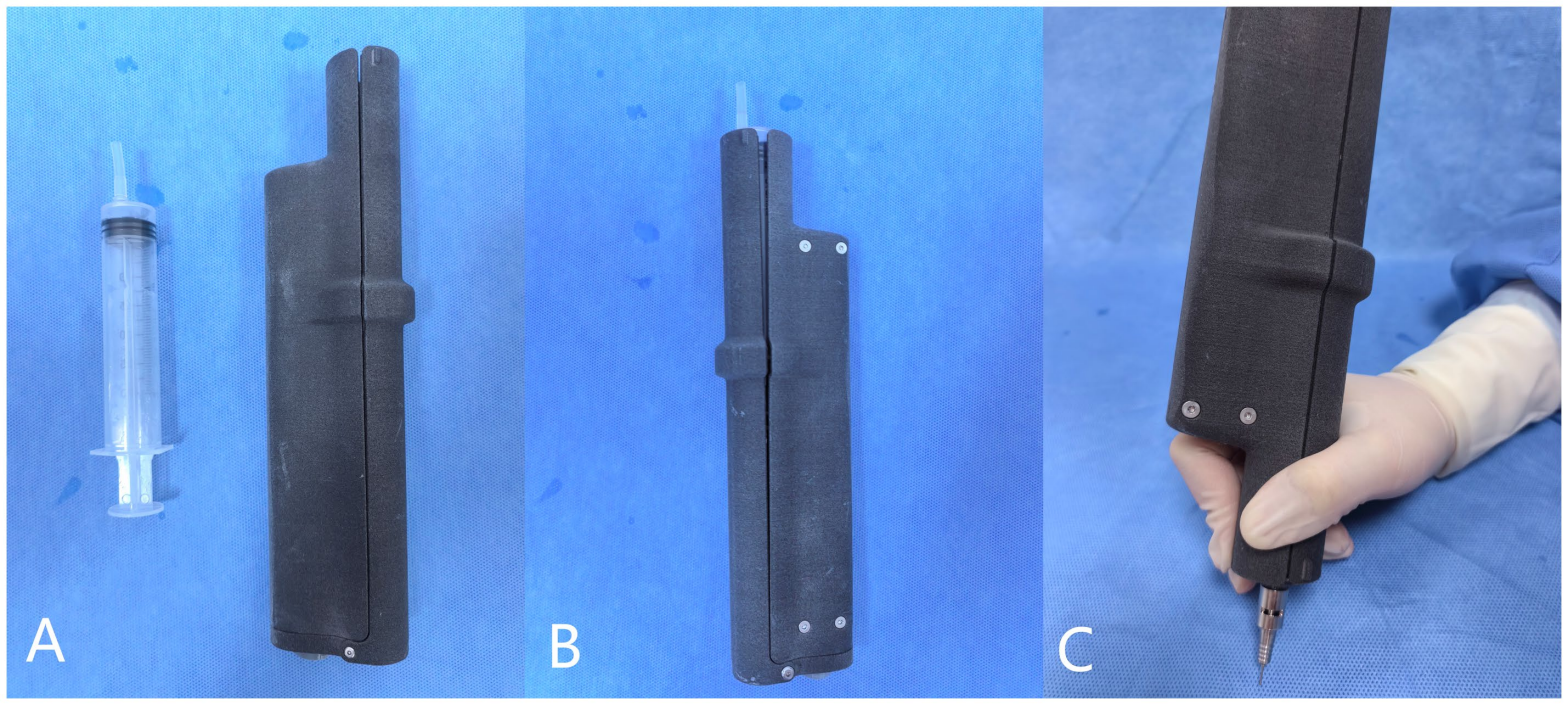
Operation using the electric device. **(A)** Electric silicone oil injector and aspirator and a sterile silicone oil syringe. **(B)** Aspiration: Sterile silicone oil syringe contained in the handheld portion, with a section of transfusion tube attached in the front. **(C)** Injection: Sterile silicone oil syringe contained in the handheld portion, with injection needle attached in the front.

In the electric group, the prototype electric silicone oil injector and aspirator was connected to a sterile silicone oil syringe. The injection rate was preset at variable 2 or 3. The injection needle was inserted through a scleral port, and silicone oil was delivered until the predefined endpoint was reached. The injection time and injected volume were then recorded (Figure 3).

**Figure 4:**
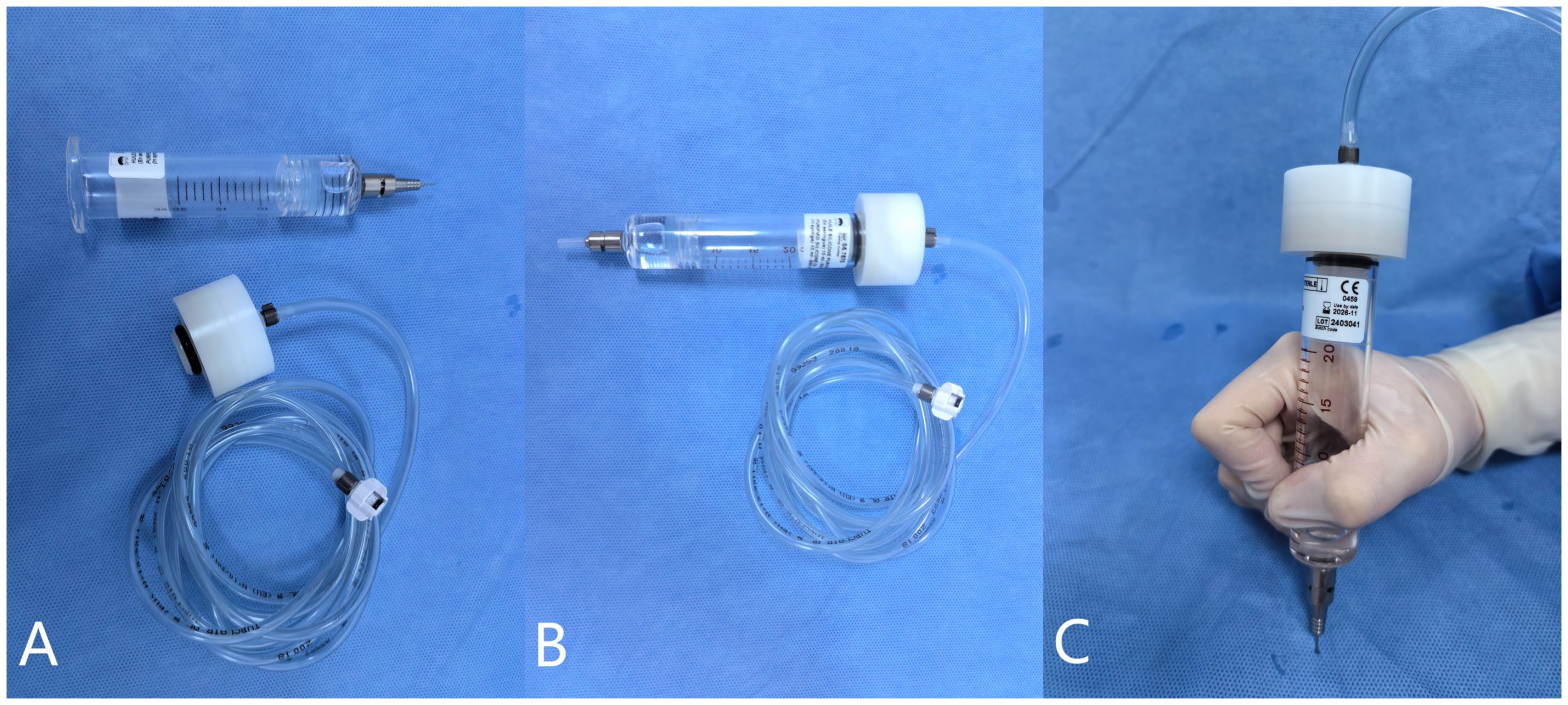
Operation with the vitrectomy machine-based system. **(A)** The dedicated pipeline of vitrectomy system and sterile silicone oil syringe. **(B)** Aspiration: Sterile silicone oil syringe connected to the pipeline, with a section of transfusion tube attached in the front. **(C)** Injection: Sterile silicone oil syringe connected to the pipeline, with injection needle attached in the front.

In the vitrectomy group, the dedicated silicone oil injection line of the vitrectomy system was connected according to the manufacturer’s standard configuration. After insertion of the injection needle through the scleral port, silicone oil injection was initiated and continued until the same predefined endpoint was reached. Injection time and injected volume were recorded (Figure 4).

**Figure 5:**
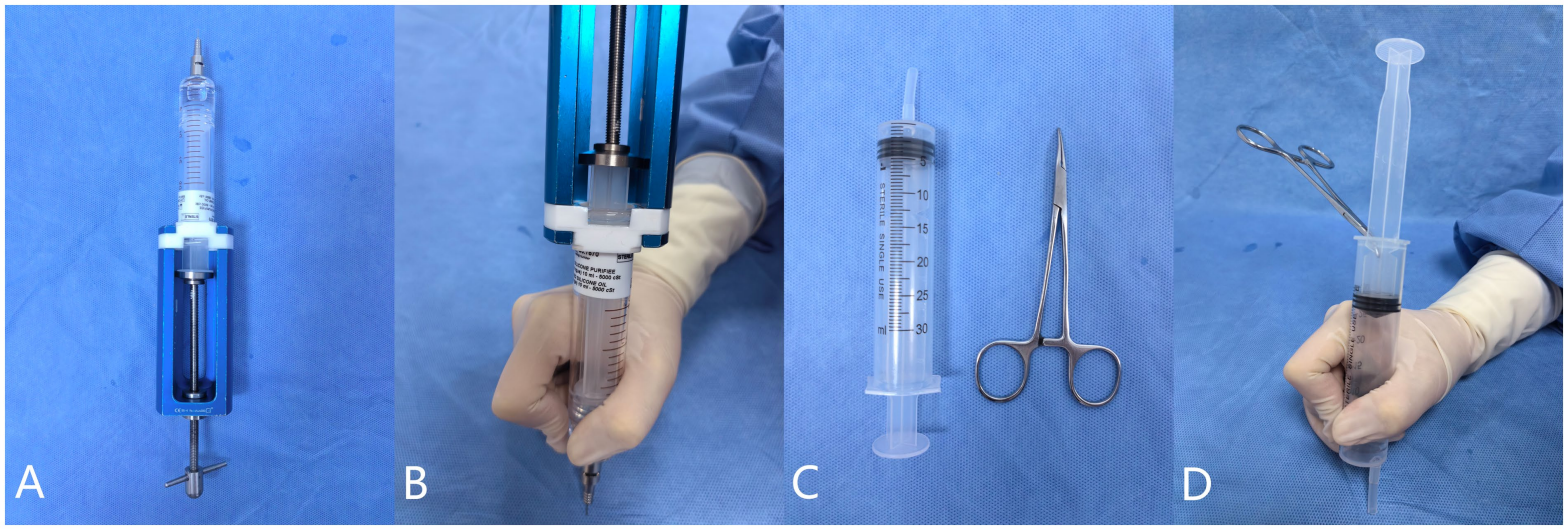
Manual injection and aspiration. **(A)** Injection: Sterile silicone oil syringe contained in the screw-driven injector, with injection needle attached in the front. **(B)** Demonstration of injection. **(C)** Aspiration: Sterile silicone oil syringe with a section of transfusion tube attached in the front, hemostatic forceps. **(D)** Demonstration of aspiration.

In the manual group, silicone oil was drawn into a syringe connected to a matched 23-gage injection needle. The surgeon manually injected the silicone oil at a steady pace as in clinical practice under microscopic visualization until the same procedural endpoint was considered to have been reached. Injection time and injected volume were recorded (Figure 5).

To improve consistency across groups, the endpoint for injection was defined as intraocular pressure (IOP) reach 20 mmHg, measuring with Schiotz tonometer. After air bubble disappeared in vitreous cavity, Schiotz tonometer was used by assistant every 5 s until endpoint. Injection was stopped once the target pressure was reached. Injection time and injected volume were then recorded for each eye.

#### 2.3.2 Silicone oil aspiration

After completion of silicone oil injection, aspiration was performed using the corresponding system assigned to each group.

In the electric group, the aspiration setting was fixed at Variable 2 or 3 (Figure 3).

In the vitrectomy group, aspiration pressure was set to 600 mmHg (Figure 4).

In the manual group, silicone oil was aspirated manually using a syringe, while vacuum pressure was maintained by hemostatic forceps (Figure 5).

In all groups, aspiration continued until no obvious residual silicone oil could be identified under the operating microscope and the aspirated volume had reached the expected endpoint for complete removal. Aspiration time and aspirated volume were recorded for each eye.

#### 2.3.3 Assessment of handling stability

Handling stability during the injection phase was evaluated by analysis of the recorded surgical videos. A square region with a side length equal to 1.5 times the external diameter of the 23-gage cannula (0.96 mm) was generated, with its center fixed at the cannula entry site. During video playback, each event in which the instrument tip or cannula entry position moved beyond the predefined square boundary was counted as one shake. The total number of shakes during the injection phase was recorded for each eye.

To reduce subjective bias, two observers who were masked to group allocation evaluated the recordings according to the same predefined rule. The final shake count for each case was calculated as the average of the two observers’ counts and was used for statistical analysis.

### 2.4 Statistical analysis

Statistical analyses were performed using SPSS version 26.0 (IBM Corp., Armonk, NY, United States). Continuous variables were first assessed for normality using the Shapiro–Wilk test and for homogeneity of variance using Levene’s test.

For variables that met the assumptions of normal distribution and homogeneity of variance, data are presented as mean ± standard deviation (SD), and intergroup comparisons were performed using one-way analysis of variance (ANOVA), followed by Tukey’s honestly significant difference (HSD) test for *post hoc* pairwise comparisons.

For variables that did not satisfy these assumptions, data are presented as median and interquartile range (IQR), and comparisons among groups were performed using the Kruskal-Wallis test. When the overall test was significant, *post hoc* pairwise comparisons were conducted using Dunn’s test with Bonferroni adjustment.

All statistical tests were two-sided, and a *p* < 0.05 was considered statistically significant.

## 3 Results

### 3.1 Comparison of silicone oil injection performance

Silicone oil injection performance was compared among the three groups in terms of injection time and injection volume (Table 1). The injection time differed significantly among the three groups (one-way ANOVA, *F* = 24.246, *p* < 0.001). Tukey HSD post hoc analysis showed that the manual group had a significantly shorter injection time (65.90 ± 15.34 s) than both the electric group (125.30 ± 29.92 s, adjusted *p* < 0.001) and the vitrectomy group (142.70 ± 29.60 s, adjusted *p* < 0.001). No significant difference in injection time was observed between the electric and vitrectomy groups (adjusted *p* = 0.305).

**Table 1:** Efficiency of three instruments during the silicone oil injection phase.

Indicators	Electric injector group (*n* = 10)	Vitrectomy group (*n* = 10)	Manual group (*n* = 10)	*F*-value	*P*-value
Injection time (s)	125.30 ± 29.92	142.70 ± 29.60	65.90 ± 15.34	24.246	<0.001
Injection volume (ml)	2.75 ± 0.63	3.05 ± 0.69	2.75 ± 0.68	0.676	0.517

**Figure 6:**
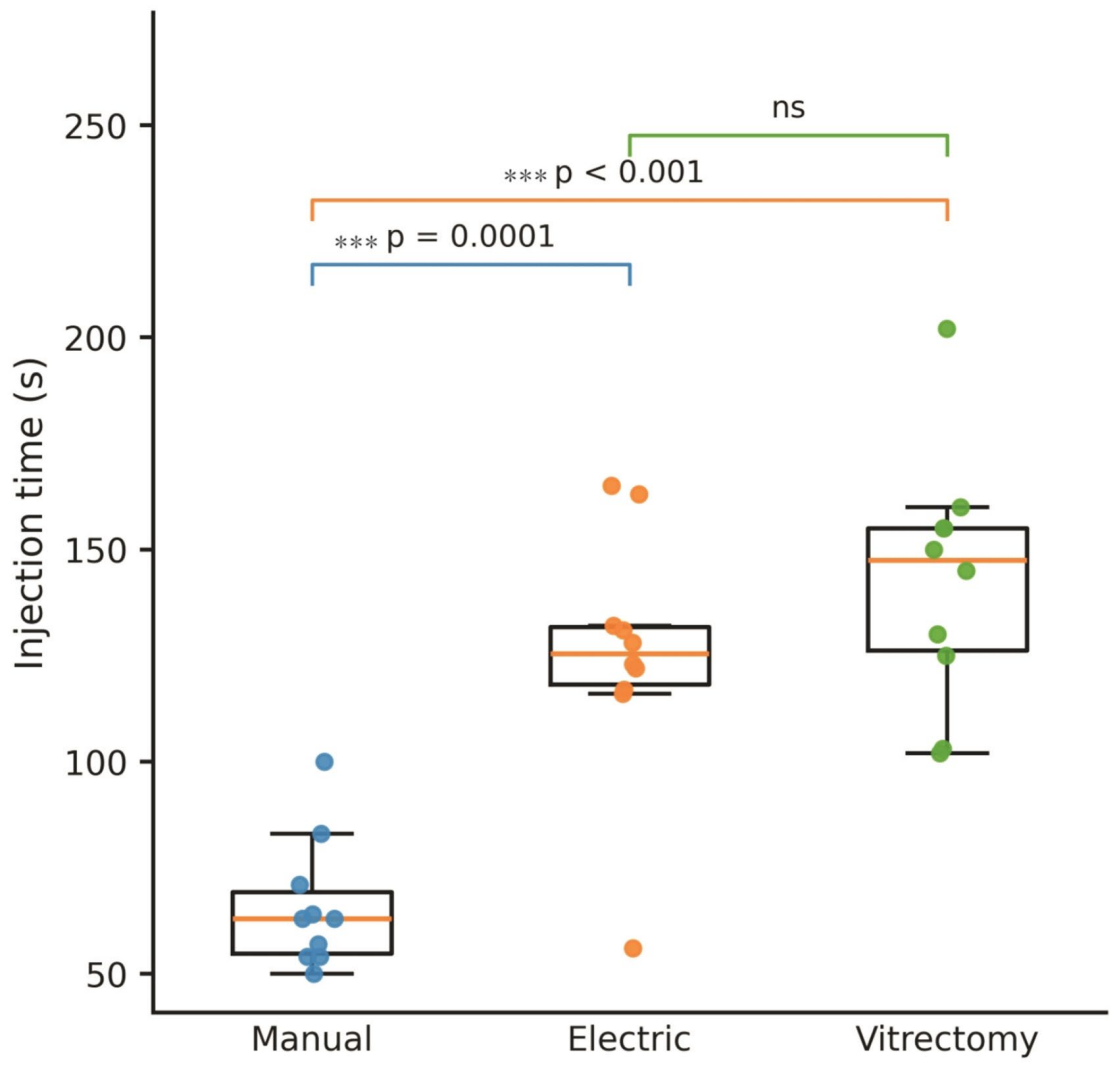
Box plot and comparisons for the injection time of the three groups. *p < 0.05, **p < 0.01, ***p < 0.001. ns: no significance.

No significant difference was found in the final injected silicone oil volume among the three groups (one-way ANOVA, *F* = 0.676, *p* = 0.517), indicating that the observed difference in injection time was not attributable to differences in final injection volume (Figure 6).

### 3.2 Comparison of silicone oil aspiration performance

**Figure 7:**
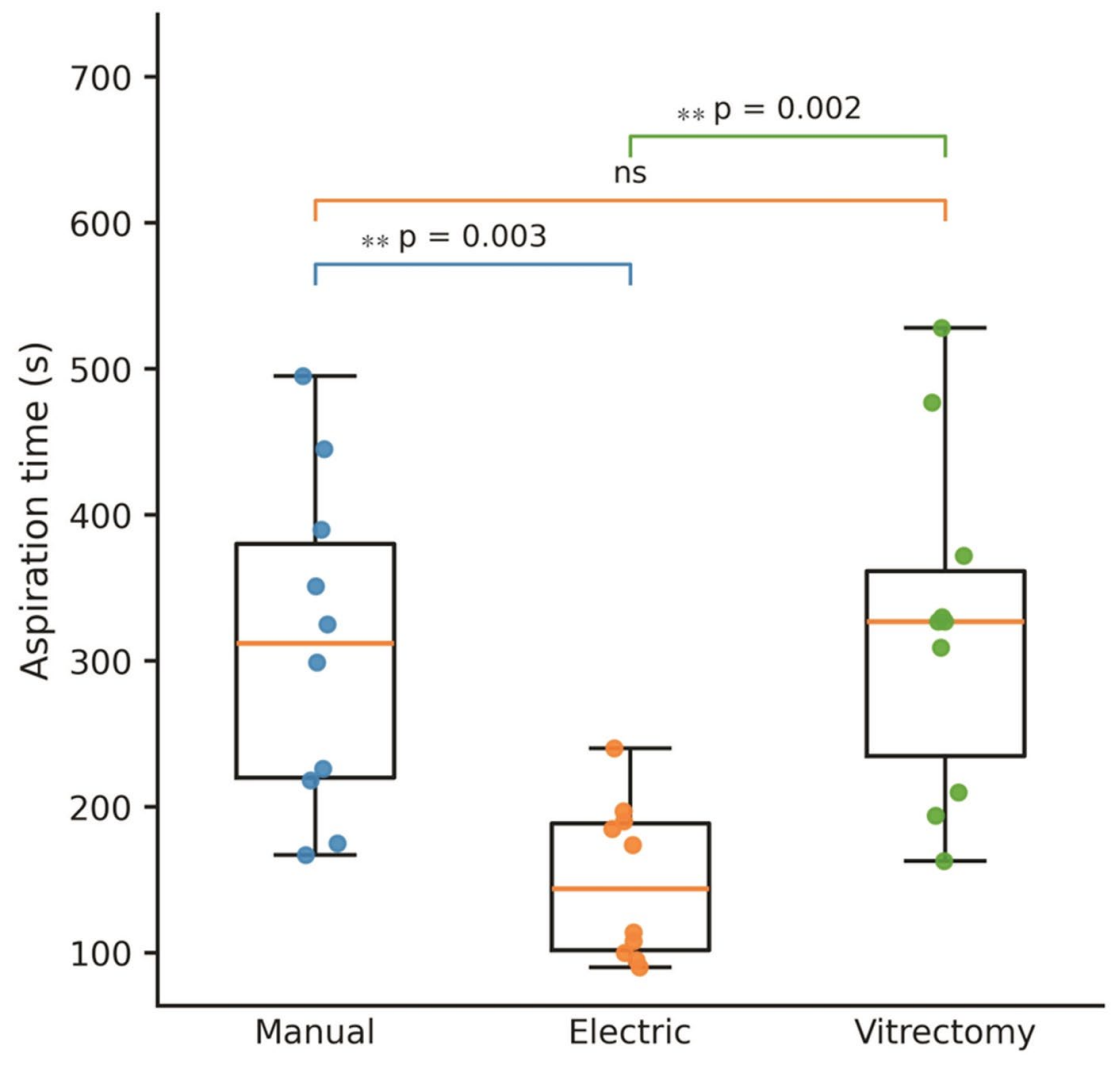
Box plot and comparisons for the aspiration time of the three groups. *p < 0.05, **p < 0.01, ***p < 0.001. ns: no significance.

Silicone oil aspiration performance was evaluated by aspiration time and aspirated volume (Table 2). Aspiration time differed significantly among the three groups (one-way ANOVA, *F* = 9.560, *p* = 0.001). Tukey HSD *post hoc* analysis showed that the electric group (149.30 ± 53.63 s) had a significantly shorter aspiration time than the vitrectomy group (323.70 ± 117.11 s, adjusted *p* = 0.0015) and the manual group (309.10 ± 113.07 s, adjusted *p* = 0.0034). No significant difference was observed between the manual and vitrectomy groups (adjusted *p* = 0.942). Aspirated volume did not differ significantly among the groups (Kruskal-Wallis test, *H* = 5.091, *p* = 0.078) (Figure 7).

**Table 2:** Efficiency of three instruments during the silicone oil aspiration phase.

Indicators	Electric injector group (*n* = 10)	Vitrectomy group (*n* = 10)	Manual syringe group (*n* = 10)	F/H value	*p*-value
Aspiration time (s)	149.30 ± 53.63	323.70 ± 117.11	309.10 ± 113.07	9.560	0.001
Aspiration volume (mL), median (IQR)	4.75 (1.75)	5.00 (1.75)	7.00 (0.75)	5.091	0.078

### 3.3 Comparison of handling stability during injection

**Figure 8:**
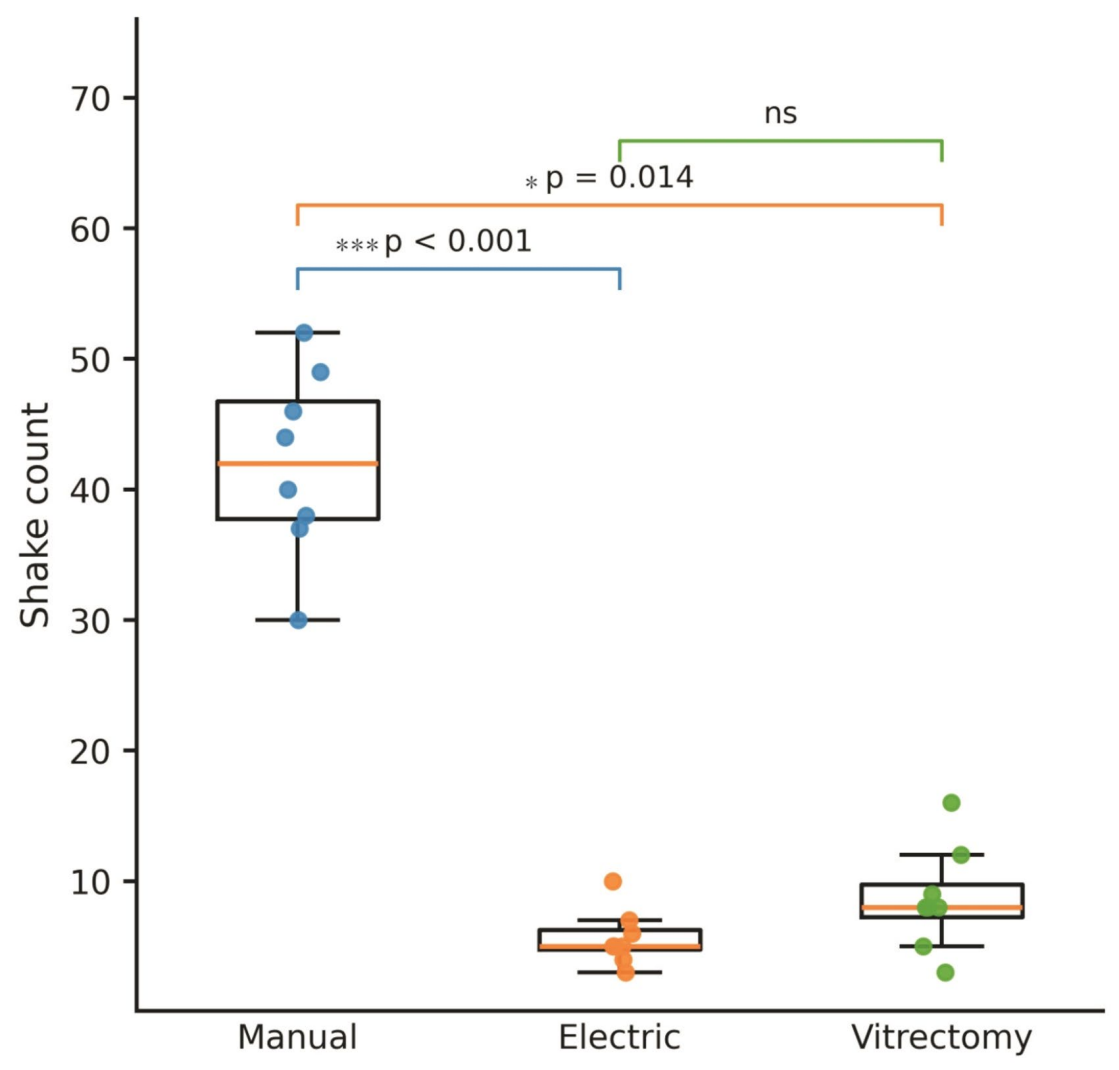
Box plot comparisons for the instrument shake number of the three groups. *p < 0.05, **p < 0.01, ***p < 0.001. ns: no significance.

Handling stability during injection was assessed by the number of instrument shakes identified on video analysis. Since the shake-count data violated the assumption of homogeneity of variance, group differences were analyzed using the Kruskal-Wallis test. A significant difference was observed among the three groups [*H*(2) = 16.749, *p* < 0.001]. Dunn’s *post-hoc* test with Bonferroni correction showed that the manual group had significantly more shakes than both the electric group (adjusted *p* < 0.001) and the vitrectomy group (adjusted *p* = 0.014), whereas no significant difference was found between the electric and vitrectomy groups (adjusted *p* = 0.769). The median shake count was 42.0 (IQR, 9.0) in the manual group, 5.0 (IQR, 1.5) in the electric group, and 8.0 (IQR, 2.5) in the vitrectomy group. These findings indicate that the handling stability of the prototype electric silicone oil injector and aspirator was comparable to that of the vitrectomy machine–based system and superior to that of manual syringe operation during the injection phase (Figure 8).

## 4 Discussion

A variety of silicone oil injection and aspiration methods are currently used in vitreoretinal surgery, including manual syringe-based techniques, modified small-gage approaches, two-port methods, direct exchange strategies, and pipeline-based systems connected to vitrectomy platforms (16–25). Because of the high viscosity of silicone oil, both injection and removal require stable and controllable manipulation. However, existing approaches still have practical limitations, including operator dependence, procedural complexity, limited consistency of pressure control, and variability in removal efficiency (18–30). Although previous studies have explored optimization of cannulas, tubing systems, and removal techniques (16–25), relatively few investigations have addressed a dedicated integrated electric system for both silicone oil injection and aspiration. In this context, the broader literature on precision ophthalmic instrumentation, sensor-assisted vitreoretinal devices, and robot-assisted microsurgery supports the feasibility of a reusable and controllable motor-assisted system for silicone oil manipulation (31–36).

The potential economic implications of the device are of interest. The handheld unit and the connecting wire were able to tolerate steam sterilization at 135 °C, allowing repeated use under standard operating room sterilization conditions. Disposable vitrectomy machine-based kits are mostly imported with price of approximately 1,000 RMB/set, which was covered by medical insurance under control policies. A reusable electric system may lower recurring disposable expenditure and reduce patients’ financial burden at the same time. Otherwise, SO aspiration can be completed by this electric device alone. This may be particularly useful in conditions like operation rooms without vitrectomy platforms. However, the present study did not perform a formal health economic analysis, and the current cost comparison should therefore be regarded as descriptive only. Any claim of cost-effectiveness would require further evaluation in studies incorporating equipment acquisition, maintenance, sterilization, consumables, workflow efficiency, and clinical outcomes (37, 38).

In the present *ex vivo* porcine eye study, we compared a prototype electric silicone oil injector and aspirator with a vitrectomy machine–based system and a manual syringe technique. The main findings were that the prototype system demonstrated handling stability comparable to that of the vitrectomy machine system and superior to that of manual syringe operation during the injection phase. Previous work in precision fundus injection, sensor-assisted vitrectomy, and robot-assisted vitreoretinal surgery has also suggested that mechatronic systems may improve motion stability and fine control compared with purely manual manipulation (31–36). There are several possible reasons for instability of manual injection. First is the heavy screw-driven injector made of stainless steel. To hold it steady, surgeons always need both hands. While in electric and vitrectomy group, this can be done easily with just one hand. The second reason is that the screw-driven injector needs an assistant or a nurse to rotate the screw, which makes it harder for holding the device stably.

Our team designed this prototype to integrate a motor-driven mechanical system, electrical actuation, and a control interface into a compact handheld platform. The rationale was to improve the controllability and consistency of silicone oil injection-aspiration while simplifying intraoperative handling. In addition, ergonomic optimization of device structure and interface may help improve usability during microsurgical procedures (34, 36). In the present study, the lower shake count observed with the electric system compared with the manual group is consistent with this design rationale. In clinical practice, the heavy, unsteady screw-driven injector can accelerate surgeon fatigue, which makes continuous operation unpleasant. In this study, both the electric group and vitrectomy group showed better surgeon satisfaction.

On the other hand, the prototype device showed favorable aspiration performance, while its injection efficiency was comparable to that of the vitrectomy machine system but lower than that of manual injection. The plastic syringe plunger would deform when force was too big. In manual group, the force was in rotation direction, this would generate continuous injecting force (while unsteady IOP might be related) even when plunger twisted. While in manual and electric group, the force was in straight line direction, injection force weakened when plunger bended. A plunger made by stiffer material might solve this problem. Otherwise, the speed of manual injection rely on the force application and lubrication of the screw. But the lubrication of the screw would decline obviously after several sterilization, which takes more strength to maintain the same performance. By contrast, the prototype electric system was designed to provide stable and adjustable actuation with the micro linear motor, which may reduce variability caused by operator technique.

As to volumes, we can find aspiration volumes vary widely (4.75–7.0 mL), while injection volumes are nearly consistent. This might be related to suction forces generated by different devices. Linear force led to regular flows, which resulted in steady outflow of SO. While nonlinear, unsteady forces led to turbulence in vitreous cavity, which resulted in outflow of SO and intraocular irrigating solution mixture. This is the possible reason for larger aspiration volume in manual group.

Several limitations should be acknowledged. First, the sample size was limited. Therefore, this study should be regarded as preliminary proof-of-concept investigation rather than a confirmatory study. Although significant differences were observed in several performance-related outcomes, particularly aspiration time and video-based shake counts, these findings should be interpreted cautiously and require validation in future studies with larger sample sizes. Second, only one silicone oil viscosity and one gage configuration were tested, which limits generalizability. Third, all procedures were performed by the same surgeon, which reduced inter-operator variability but did not allow assessment of performance across users with different levels of experience. Forth, it would be better if electric and manual group could match specific rates or pressures. In this study, Variable 4–5 in electric group produced force too large that would bend the syringe plunger, so Variable 2–3 were used. While considering the discrepancy between different operators, it is harder to set a quantitative parameter for manual group. Finally, this was an *ex vivo* porcine eye study, and such a model cannot fully reproduce the physiological conditions of living eyes, including dynamic intraocular pressure regulation, tissue perfusion, inflammatory response, wound behavior, and intraoperative patient-related variability (31, 33, 35, 39, 40).

These limitations also define the priorities for future work. Comparative evaluation under more closely matched operating parameters would also strengthen interpretation of group differences. In addition, studies involving different silicone oil viscosities, instrument gages, and surgical scenarios may provide a more comprehensive understanding of device performance (16–25). If confirmed in subsequent studies, the prototype electric system may have potential as an alternative to existing approaches for silicone oil injection-aspiration in clinical practice.

In summary, this *ex vivo* study suggests that the reusable prototype electric silicone oil injector and aspirator demonstrated good aspiration efficiency and operational stability. Although its injection efficiency was lower than that of the manual method, it was comparable to the vitrectomy machine-based system, with potential cost advantages in clinical settings. Given the *ex vivo* design, limited sample size, and absence of direct clinical outcome measures, these findings should be interpreted as preliminary and hypothesis-generating rather than confirmatory.

## Data Availability

The raw data supporting the conclusions of this article will be made available by the authors, without undue reservation.
